# Editorial Comment to Current Landscape of Urological Surgical Training: A Needs Assessment Survey in Japan

**DOI:** 10.1111/iju.70071

**Published:** 2025-04-17

**Authors:** Tomokazu Kimura

**Affiliations:** ^1^ Department of Urology Nagoya University Graduate School of Medicine Nagoya Japan; ^2^ Center for Medical Education Nagoya University Hospital Nagoya Japan

**Keywords:** faculty development, surgical education, urological training

The training of highly skilled urologists has become a pressing priority in Japan's rapidly aging society. Japanese urologists perform both surgical and medical treatments, but the transmission of surgical skills—especially for highly invasive procedures—is critical. This study provides timely insights into urological surgical training across all three medical universities in Hokkaido, a vast and diverse region of Japan, making a significant contribution to surgical education. As the traditional “See one, Do one, Teach one” approach is increasingly replaced by competency‐based training models, the authors conducted a comprehensive survey of trainees and instructors across multiple institutions to assess training practices and perceived educational disparities [1].

A key strength of this study is its high survey response rate, which enhances data reliability and reflects the growing interest in structured surgical education. The study examines various aspects, including available training resources, on‐the‐job (OJT) and off‐the‐job (Off‐JT) training methods, trainee assessment strategies, and differences in perception between trainees and instructors, offering a comprehensive overview of the current training landscape. The findings highlight both strengths and areas for improvement in Japan's urological training system. While OJT remains the primary method for skill acquisition, access to Off‐JT resources—such as laparoscopic dry labs and Virtual Reality simulators for robotic‐assisted surgery—is limited to only a few institutions. The study also discusses how infrastructure limitations and instructors' heavy workloads restrict surgical education. Furthermore, a notable gap exists between trainees and instructors: trainees often feel their surgical training is insufficient, whereas instructors believe they are providing adequate guidance. Clinical education tends to be short‐term and ad hoc, with limited stepwise assessment and evaluation, underscoring key challenges in the field.

Achieving surgical proficiency within a standard urology residency is difficult, necessitating lifelong learning. Academic societies, including the Japanese Medical Specialty Board and the Japanese Urological Association, should work toward developing integrated surgical training curricula and structured instructor development programs, as well as faculty development (FD) programs of clinical training instructors for medical residents. These efforts would enhance teaching capabilities, create educational opportunities, and improve the overall quality of urological training.

Surgical education organizations, such as the Association for Surgical Education [2] in the U.S. and the Japanese Society for Surgical Education [3], have gained attention in recent years. In Japan, the Surgeons as Educators [4] offer FD programs and instructor training workshops focused on essential teaching skills. It is time for urologists to actively engage in discussions on enhancing their educational methods. The insights from this study can serve as a catalyst for developing more effective and structured training programs, ultimately improving both urological education and patient care in Japan.

## Author Contributions

The author takes full responsibility for this article.

## Conflicts of Interest

The author declares no conflicts of interest.
